# “Pepper”: Different Spices, One Name—Analysis of Sensory and Biological Aspects

**DOI:** 10.3390/molecules30091891

**Published:** 2025-04-24

**Authors:** Pierina Díaz-Guerrero, Sofia Panzani, Chiara Sanmartin, Chiara Muntoni, Isabella Taglieri, Francesca Venturi

**Affiliations:** 1 Department of Agriculture, Food and Environment, University of Pisa, Via del Borghetto 80, I-56124 Pisa, Italy; pierina.guerrero@phd.unipi.it (P.D.-G.); sofia.panzani@phd.unipi.it (S.P.); 29690001@studenti.unipi.it (C.M.); francesca.venturi@unipi.it (F.V.); 2 Interdepartmental Research Centre “Nutraceuticals and Food for Health”, University of Pisa, Via del Borghetto 80, I-56124 Pisa, Italy

**Keywords:** SWOT analysis, *Piper nigrum* L., *Piper cubeba* L.f., *Piper longum* L., *Schinus terebinthifolius* Raddi, *Pimenta dioica* L. Merrill, *Zanthoxylum piperitum* DC.

## Abstract

Spices are a part of modern and ancient cultures due to their recognized culinary and medicinal properties. Pepper is commonly used in many recipes; however, in the field of gastronomy, the term “pepper” usually refers to a group that includes several different spices, such as black pepper (*Piper nigrum* L.), cubeb pepper (*Piper cubeba* L.f.), long pepper (*Piper longum* L.), pink pepper (*Schinus terebinthifolius* Raddi), allspice (*Pimenta dioica* L. Merrill), and Japanese pepper (*Zanthoxylum piperitum* DC.). Despite the extensive study of the chemical characterization and medicinal and culinary properties of “pepper”, sensory analysis (color, aroma profile, odor profile, and chemesthesis) of these spices have not been completed. Therefore, the aim of this review was to identify the strengths, weaknesses, opportunities, and threats within the spice supply chain considering these six “peppers” to analyze their positive and negative aspects. Finally, we selected the most representative molecules and properties of spices referred to as “pepper” to expand the research focus and highlight their key aspects related to health and sensory science for future applications. In this sense, this review provides a new strategic guideline that will help us understand and assess the key internal and external factors of pepper, allowing them to be applied in different sectors with different approaches.

## 1. Introduction

Spices have historically played a vital role in both traditional medicine and food preservation. The sacred Hindu script “Rigveda” offers the oldest documented evidence (6000 BC) of the culinary and medical uses of spices. The United States Department of Agriculture (USDA) defines spices and herbs as plant-derived substances that add flavor to any dish [1,2]. The seeds, leaves, roots, bark, flowers, and fruit are considered spices, while the leafy parts of the plant are herbs [1,2]. Spices are usually used in various forms, such as whole, ground, powdered, as pastes, and roasted [3]. Spices are rich in bioactive compounds, such as polyphenols, alkaloids, carotenoids, and capsaicinoids, which are all known for their antioxidant, antimicrobial, anti-inflammatory, and anticancer properties. The presence and concentration of such compounds in plants are related to internal and external factors. Good examples of these include the variety and ripening stages and geographic distribution of plants, as well as the extraction methods and detection techniques employed in collecting them [4]. Bioactive compounds from spices are used as natural antioxidants in beauty products and for food preservation, as flavor agents for food seasoning, as natural dyes, etc. [5,6,7,8,9].

The important and widely used products obtained from spices include essential oils (EOs), non-nutritive lipophilic secondary metabolites produced by plants. Some of these EOs exhibit bioactive properties due to their chemical compositions. Therefore, under several disciplines, researchers are investigating their possible applications in food products, considering that the maximum concentration of EOs allowed in food is around 0.025% [10,11,12]. The composition of EOs largely affects the sensory properties of spices, particularly relating to smell characteristics. However, the sensory traits of spices are also influenced by other compounds and can interact with taste receptors or induce chemesthetic perceptions. The organoleptic characteristics of spices are thus used in the gastronomy field to enhance dishes and to improve the foods’ sensory profiles [1,10,12]. In this context, pepper is widely used in a large number of recipes; additionally, it is important to clarify that, in gastronomy, the term “pepper” generally refers to a group of different spices, such as the following: black pepper (*Piper nigrum* L.), cubeb pepper (*Piper cubeba* L.f), long pepper (*Piper longum* L.), pink pepper (*Schinus terebinthifolius* Raddi), allspice (*Pimenta dioica* L. Merrill), and Japanese pepper (*Zanthoxylum piperitum* DC.). Despite extensive studies on the chemical characterization and medicinal and culinary properties of “pepper”, sensory analyses (color, aroma profile, odor profile, and chemesthesis) of these spices have not been fully performed. Some research groups are starting to explore this field with an innovative approach [13]. However, there are still few sensory studies conducted on spices in their natural forms, and none specifically on the spices analyzed in this review [14,15]. Therefore, the aim of this review was to identify and analyze the strengths, weaknesses, opportunities, and threats within the spice supply chain by applying a SWOT analysis to the six peppers listed previously, so as to analyze their positive and negative aspects, considering both the intrinsic characteristics of the raw material and external factors like the production process. In order to avoid confusion, spices belonging to the *Capsicum* genus, commonly known as “chili peppers” or “hot peppers”, were not included in this review. Finally, we selected the most representative molecules and properties of spices referred to as “pepper” to expand the research focus and highlight their key aspects in health and sensory science for future applications. To the best of our knowledge, this is the first time a SWOT analysis has been applied to spices.

## 2. The Characteristics of the Six Pepper Spices

In Table 1, the spices selected for this review are listed and are reported with their botanical and family names, geographical distributions, common names in English, French, Spanish, German, and Italian, edible organs, and methods of production.

Beyond their numerous uses in cooking and food technologies as flavoring, as well as preservative agents [9,12,23,24], as widely reported in the literature [4,12,25,26,27,28], these spices have been studied due to their high antioxidant activity and their beneficial effects on human health (Table 2).

Antioxidants derived from spices encompass a large group of bioactive compounds, including flavonoids, phenolic compounds, tannins, alkaloids, terpenes, etc., exhibiting varying levels of antioxidant activity. Further relevant studies regarding the use and dosage of the selected spices are reported in Supplementary Table S1.

The bioactive, chemical, and sensory properties of spices are highly variable and depend on multiple factors, including cultivation practices and the growing environment [71]. These factors are often shaped by societal culture, such as farmers’ beliefs regarding their agricultural methods, and are closely related to the geographical origin of the spice [72]. It is challenging to obtain detailed information on the specific production methods of spices, primarily due to the reliance on traditional, empirical techniques that vary significantly across different regions. These methods are often passed down through generations without formal documentation, making it difficult to standardize or study them in depth. This diversity complicates the efforts to assess or compare the quality, composition, and potential health effects of spices produced in different locations [28,73].

### 2.1. Strengths of the Six Pepper Spices

#### 2.1.1. Chemosensory Characteristics

The addition of spices, rich in aromatic and chemesthetic compounds, increases the sensory impact of the food preparations. Spices contain a range of chemosensory-active compounds (CaCs) that interact with sensory receptors (smell and taste) but also chemesthetic compounds that elicit distinct sensations, such as pungency, tingling, numbing, warming, etc.

The CaCs add complexity to dishes, stimulating taste receptors and olfactory senses in ways that enhance both the flavor and appeal of foods. These characteristics are appreciated by consumers who are searching for natural alternatives to reduce the consumption of salt, sugar, and fat. The high concentration of CaCs and their variability across different spices must be carefully considered in relation to the food being processed. Therefore, it is essential to assess the sensory effects of each spice by itself and combined with different food matrices.

The selected species are well known for their wide contents in various CaCs (see the following Section 3.1, Section 3.2, Section 3.3, Section 3.4, Section 3.5 and Section 3.6), and among them, piperine (*Piper nigrum* L., *P. cubeba* L.f., and *P. longum* L.) [74] and sanshool compounds (*Zanthoxylum piperitum* DC.) are renowned for their multi-faceted sensory activities, since they can stimulate both taste and smell, also eliciting chemesthetic sensations.

Piperine is the compound responsible for the pungent pepper-like flavors of *Piper nigrum* L., *P. cubeba* L.f., and *P. longum* L., promoting pronounced salivation and numbness sensations (chemesthetic threshold level (CTL) = 3.0 nmol/cm^2^) [25,30,44]. As a chemical irritant, piperine stimulates the trigeminal nerve in the oral cavity and interferes with/stimulates taste and odor. Some authors [75] reported that piperine increases saltiness perception, but it also increases bitterness and decreases sweetness. According to the literature, piperine content is present in a wide range of concentrations in *P. nigrum* L. (2–9%), *P. cubeba* L.f. (~0.1%), and *P. longum* L. (3–5%); therefore, it is very complex to predict the chemosensory activities of these plants, considering the sensory threshold levels [76,77,78,79,80,81,82,83].

The sanshool class is responsible for the pungent, tingling, and numbing flavor in *Zanthoxylum piperitum* DC.; meanwhile, geraniol, linalool, citronellal, methyl cinnamate, piperitone, and trans-anethole are responsible for its minty, sweet, anise, and licorice aroma [40,41,84,85]. Regarding the sanshool compounds, the alkylamide α-sanshool (CTL = 13 μg/mL) induces burning, tingling, and numbness sensations, and its content in the *Z. piperitum* DC. ranges between 4.7 and 12 mg/g DW. The alkylamide β-sanshool (CTL = 14 μg/mL) causes tingling and a bitter taste (0.1–1.1 mg/g DW); the hydroxyl-α-sanshool (CTL = 38 μg/mL) is associated with burning and tingling sensations (20–52 mg/g DW); the hydroxyl-β-sanshool (CTL = 78 μg/mL) is responsible for numbness, astringency, and a bitter taste (0.6–5.6 mg/g DW) [86]. The content in *Z. piperitum* DC. is highly variable, making it extremely challenging to accurately estimate its sensory profile based on data from the literature alone without conducting an actual sensory evaluation.

Another interesting CaC that is associated with the innocuous warming oral sensation of *Pimenta dioica* L. Merrill and *Piper cubeba* L.f. is eugenol, which constitutes 60–90% of the EO in the former and 34% in the latter, respectively; however, to the best of our knowledge, the CTL for this compound has not been reported in the literature. Eugenol is also a well-known odorant associated with spicy, smoky, and herbal scents (odor threshold in water = 6–30 ppb) [87].

According to the literature, the pungent, bitter, and astringent flavors of *Schinus terebinthifolius* Raddi are associated with terpenes and phenolic compounds [27,88]. *S. terebinthifolius* Raddi is naturally characterized by a volatile fraction rich in terpenes, the primary ones being alpha-pinene, alpha-phellandrene, and limonene, which impart spicy, balsamic, and floral notes. Regarding the phenolic fraction, the main compounds identified belong to the phenolic acid class (gallic acid, syringic acid), flavanones (naringenin, hesperidin), and proanthocyanidins (procyanidin A2, B1, and B2).

#### 2.1.2. Multi-Faceted Bioactivity

Spices are rich in secondary metabolites that can positively interact with living organisms by improving certain aspects of metabolism and preventing specific diseases, thereby promoting health. The most common biological activities of spices include their antioxidant and antimicrobial activities attributed to flavonoids, their anti-inflammatory action linked to terpenoids, and their digestive benefits associated with alkaloids [89,90,91].

In particular, the spices considered in this review are highly valued medicinal plants that have been widely utilized in traditional and folk medicine for centuries. Renowned for their therapeutic versatility, these plants are used to address a variety of health issues, including digestive disorders, respiratory diseases, and inflammatory conditions. This highlights their valued role in natural healing practices worldwide [25,49,55,57,92,93].

Based on the data reported in the literature, there is significant heterogeneity in terms of the biological effects observed as associated with the spices under consideration. However, it is challenging to obtain a clear overall picture, as the studies reported in the literature often refer to effects associated with different types of extracts, sometimes addressing essential oils, and in some cases individual compounds. Additionally, there is substantial variability in the research methodologies, which include in vivo or in vitro tests performed on different target organisms.

Furthermore, the number of studies reporting data on the effects associated with the consumption of the spice in its natural form is very limited. The representativeness of these studies is further reduced by the significant variability in the spice’s composition and, in particular, the wide variability in the concentrations of bioactive compounds.

Therefore, further studies are needed that seek to understand the bioactive properties of these spices in order to facilitate the creation of innovative therapeutic techniques that leverage compounds with precise biological effects.

The main bioactive effects on the different apparatuses associated with the spices analyzed in this review are summarized in Table 2. In the Supplementary Materials Table S1 gives an overview of relevant studies regarding the spices and the uses and dosages of their EOs/extracts/compounds, along with the principal results. Some of the main secondary metabolites of interest for each spice will be discussed, along with their bioactive effects, throughout the review.

#### 2.1.3. Natural Additive for Food Preservation

Essential oils (EOs) and spice extracts are widely used as natural food preservatives due to their chemical composition (flavonoids, terpenoids, curcumins, lignans, carotenoids, saponins, phytosterols)—for instance, their high phenolic compound contents. Within this class, the flavonoid ones present antioxidant, antifungal, antibacterial, and antiviral activities and are thus commonly added into foods to impart these properties [8,9,24,94]. A good example of an aromatic compound with interesting preservative properties is eugenol, which is largely used as a food additive not only for its flavoring activity but also for its antimicrobial and antioxidant activities [95].

EOs and spice extracts can be used in food technology to add value and improve the stability of foods, especially when they are particularly susceptible to pests, pathogens, and oxidative degradation.

Due to their preservative properties, EOs and spice extracts can be advantageous, as in the current era, the food industry is focused on finding natural methods with potential preservative effects to reduce the use of synthetic additives [8,12,78]. In this context, it might be interesting to consider the direct use of spices as additives. For example, *Piper nigrum* L. has historically been used as a preservative due to its antimicrobial and antioxidant properties. However, its high compositional variability makes it difficult to quantify the spice’s effectiveness and, consequently, to determine the appropriate dosage to achieve a significant benefit without compromising the sensory profile of the product.

The direct incorporation of EOs into food formulations may be less effective, probably because they start to degrade quickly as a result of interactions between their unstable, volatile compositions and external factors (i.e., light, oxidation, heating). Recent works have developed new systems to improve the stability of EOs by encapsulating them in liposomes, polymeric particles, and solid lipid nanoparticles [96,97].

Some researchers have successfully carried out tests on the use of spice EOs as natural agents in active packaging to release their compounds into the food over time [8,12,98]. Spice EOs are thus used as additives in biodegradable or edible films and as coatings in active food packaging in order to extend shelf life and add value to the product. The most important actions of EOs are the inhibition of micro-organisms, which reduces the phenomenon of lipid oxidation in food, and pest contamination [8,12,98]; some authors [99,100] have also reported improvements in the physical and chemical properties of the film as a result.

### 2.2. Weaknesses of the Six Pepper Spices

#### 2.2.1. Instability of Aromatic Compounds

One of the challenges related to spices is their storage. The principal components of spices are essential oils and aromatic compounds, which, due to their chemical structure, are hypersensitive to external factors, such as humidity, temperature, and light exposure. According to the literature, the EO concentration decreases when a spice is subjected to a drying process due to the loss of monoterpenes [101]. The main EO constituents can be classified as lipophilic terpenoids, phenylpropanoids, or short-chain aliphatic hydrocarbon derivatives of low molecular weight. EOs undergo oxidation, cyclization, isomerization, or dehydrogenation reactions, triggered by enzymatic, chemical, or physical processes. The stability of EOs is also influenced by plant health, growth stage, soil and climatic factors, and time of harvest. The degradation of EOs reduces the intensity and taste of the compounds; moreover, it may affect their bioactivity [12,102].

Therefore, careful handling, storage, and processing are essential to preserve the quality, efficacy, and sensory characteristics of aromatic compounds in various applications.

#### 2.2.2. Long Supply Chain

Spice production involves a long supply chain in which spices must first undergo local pretreatment before usually being delivered to another continent for the final stages of production [21,103]. This procedure can be difficult and end up compromising the quality of the product. If the spices are not well fixed at the manufacturing site, they may deteriorate during transport due to temperature and humidity changes, which could cause safety problems due to the possible proliferation of pathogenic micro-organisms. Therefore, special care must be taken in the packaging of pretreated spices, and the materials must have adequate gas permeability or moisture barrier properties. The raw material received by the purchaser may not be suitable for final processing, which entails a considerable waste of resources.

### 2.3. Opportunities of the Six Pepper Spices

Consumer awareness has increased in recent years, demanding the substitution of synthetic additives, such as synthetic antioxidants, for natural additives. Since the chronic effect of the consumption of these synthetic additives, as well as the health risk it represents, is uncertain, the search for plant-based resources is a current trend related to food quality. EOs and plant extracts are the plant-based products most commonly used in active food packing, increasing the shelf life and adding value to the product [8,94,104].

The polysaccharides present in *Schinus terebinthifolius* Raddi can be used as stabilizers, gelling and foaming agents to maintain or improve food texture [34].

Some spices’ essential oils can present a pungent odor, such as the EOs of *Pimenta dioica* L. Merrill; therefore, to reap the benefits of its bioactive properties without risking consumer rejection, the use of the technology of nanoencapsulation is suggested to mask the intense aroma [12]. Encapsulation techniques also protect EOs against external factors [105].

### 2.4. Threats of the Six Pepper Spices

#### 2.4.1. Impactful Chemosensory Characteristics

As reported in Section 2.1.1, the spices under consideration are characterized by the presence of CaCs, which play a key role in defining their sensory profiles. In this context, compounds with chemesthetic activity (e.g., β-caryophyllene, piperine, eugenol, sanshool compounds, procyanidins, etc.) are responsible for certain sensations, such as astringency, tingling, numbing, buzzing, burning, and cooling in the mouth, which are not always appreciated by consumers. Additionally, their high contents of essential oils, which are typically rich in compounds with low olfactory thresholds and limited stability under oxidation, further complicate their use. Furthermore, the presence of CaCs in highly variable concentrations, even within the same species, makes it challenging to predict their sensory profile. These factors make it difficult to use these spices as additives, whether in food formulations or as components of bioactive packaging, for example.

#### 2.4.2. Adverse Reactions

Spices are derived from plants and may therefore cause adverse reactions in some people. These reactions can range from mild allergic reactions, such as skin irritation or mild respiratory problems, to more serious systemic reactions that can affect several body systems [68,106,107].

Piperine from *Piper nigrum* L. showed embryotoxic effects in rats, along with the disturbance of spermatogenesis and maternal reproduction, while in humans, it underwent interactions with medical products at the maximum dose per day of 14 mg. In this instance, the consumption of *P. nigrum* L. may affect pregnant or breastfeeding women [25,108]. The use of high concentrations of piperine can be toxic to the central nervous and reproductive systems [79].

Excessive doses of *Piper cubeba* L.f. berries may cause burning pain, nausea, griping, vomiting, and purging effects [30]; in addition, *Piper cubeba* L.f. exhibits oxytocic effects, being harmful to women during pregnancy [109].

The (-) cubebin compound present in *Piper cubeba* L.f. promotes cytotoxicity in rats at 280 μm after 24, 48, and 72 h [64]. Some lignans of *Piper cubeba* L.f. exhibit inhibitory activity against cytochrome P450 enzymes, which are a key factor in the metabolism of many commonly used drugs, and so, this may lead to drug interactions [109]. The stem bark decoction of *Schinus terebinthifolius* Raddi may represent a health risk due to its mutagenic properties and capacity to cause DNA damage in bacteria; also, the use of *Schinus terebinthifolius* Raddi for topical wounds may present a health risk [45,110]. Further toxicological studies are needed.

Understanding the potential for these adverse effects is crucial, especially as spices become more widely used in different diets and cuisines. The awareness and proper identification of specific spices that may cause sensitivities can help to effectively manage and mitigate these risks.

Regarding toxicology studies of spices, to the best of our knowledge, there are no previous articles that have studied the potential toxic effects of the whole spice. Instead, some articles [26,41,64,108,111] have reported on the toxicity evaluation of single components with potential harmful or irritant effects.

However, since an individual compound does not predict or determine the potential toxicity of the whole spice, we cannot draw a definitive conclusion on this aspect. In this sense, we encourage researchers to conduct further toxicological studies on the most common modes of use of the spice (whole and ground) in order to determine the optimal dose to reap the benefits of the spice without adverse effects.

#### 2.4.3. Interaction with Drugs

The high presence of bioactive compounds can, in some cases, represent a threat to the consumer. In fact, some compounds can interact with the enzymatic mechanisms responsible for the metabolism of drugs, resulting in their increased or decreased absorption [109,112]. Piperine can influence the bioavailability of some drugs due to its interaction with the enzymes responsible for drug metabolism, and/or it may inhibit the drug transporter or the efflux pumps; in many cases, this can impact the bioavailability and the efficacy of the drug [108,113].

## 3. Key Molecules and Properties of Selected “Peppers”

### 3.1. Black Pepper (Piper nigrum *L.*)

The *Piper* genus is the largest of the Piperaceae family, with about two thousand species of *Piper* having been reported [19,114]. Depending on the treatment applied to the peppercorns during harvesting, different types of pepper are obtained. Black pepper is the most famous *Piper,* and it is classified as *Piper nigrum* L., along with white pepper, green pepper, and Kampot pepper. Black pepper is produced by harvesting the fruit when it is partially ripe and yellow before drying it until it acquires its characteristic black color and wrinkled appearance [18,115]. India is the main producer of *P. nigrum,* with 51 cultivars with major distribution in Hainan, Yunnan, and Guangdong in China and in Europe [25,115]. Moreover, black pepper is well known for its richness in phytochemical compounds [25]. The most important classes of compounds isolated from *Piper nigrum* L. are alkaloids and amides, while piperine is the most abundant compound [25,44,108]. There are also phenolic compounds, volatile oils, lignans, phenylpropanoids, terpenes, chalcones, steroids, and coumarins [55]. According to the literature [25], 31 biological activities have been associated with black pepper, such as antioxidant, antidiabetic, analgesic, anticancer, and anti-inflammatory activities.

The essential oil in black pepper berries constitutes 0.4–7% of the dry weight [78,116], and it is known that many of the compounds present in the EOs are responsible for their flavor profiles. The optimum pepper aroma (“top peppery note”) is achieved when the monoterpene content (excluding α- and β-pinene) is high and the pinene content is low. This results in a pungent fruity aroma with piney, citrusy, herbaceous, woody, earthy, and spicy notes. The final flavor profile of black pepper is also influenced by various factors, such as the age of the peppercorns, whether they have been freshly ground, the shelf life, and the matrix in which they are used (water-, fat-, or starch- based) [82]. Table 2 and Table S1 summarize the main biological activities, along with relevant studies assessing black pepper use and dosage, and the principal results.

**Figure 1:**
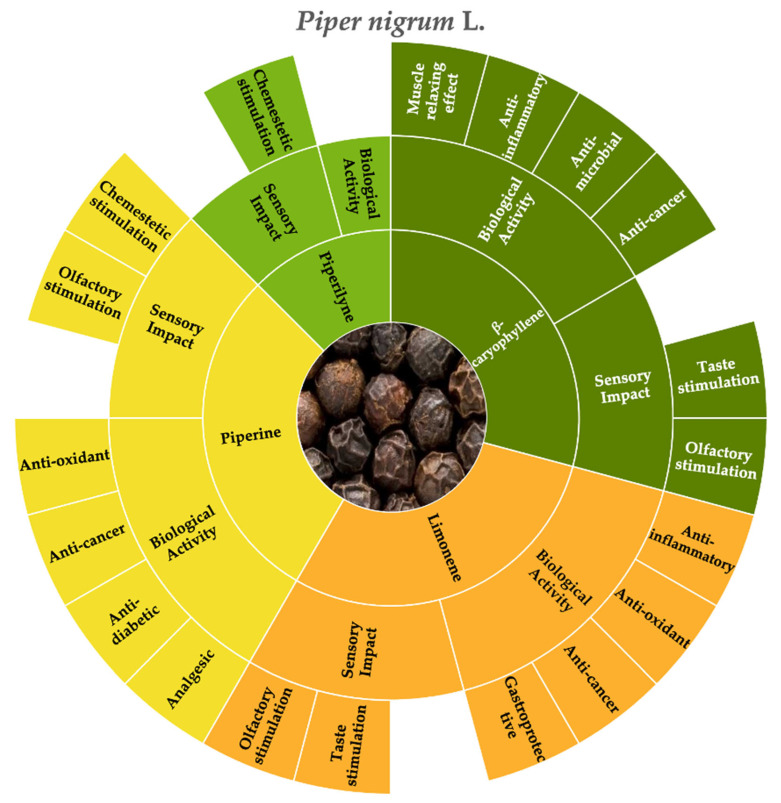
Key molecules and information gaps related to the sensory stimulation and biological activity of black pepper (*Piper nigrum* L.).

According to the consulted literature, the molecules that most affect both the chemosensory and biological characteristics of black pepper are piperine, piperyline, limonene, and β-caryophyllene (Figure 1).

Piperine belongs to the alkylamide class. Its content in black pepper fruits is approximately 2–7%, while sometimes reaching 9% [78,79,80,81,82,83]. This compound is associated with many sensory and biological activities. Regarding the sensory traits, piperine is the major compound responsible for the pungency of black pepper, with a threshold level of 3.0 nmol/cm^2^ [74]. It can activate the transient receptor potential vanilloid (TRPV1), the same as capsaicin [116,117,118], and TRPA1 [118], which are both present in the mouth, and when activated, they give rise to an excitatory current in the sensory neurons [119]. It is not yet fully understood how piperine succeeds in activating the TPRV1 receptor [117]. This sensory reaction related to the pungent compounds in pepper, however, is not actually a thermal sensation, even though the term “hot” is used to describe it. It is more accurately classified as a form of chemically induced irritation, distinct from taste and touch [82]. Piperine can stimulate the olfactory receptors correlated with odor descriptors such as peppery, spicy, and animal [120].

With regard to biological activities, the most important is the antioxidant one; this is related to the chemical structure of the compound, and it can stabilize small reactive radical species while is also being able to inhibit lipid peroxidation [74,121,122]. Treatment with piperine can modulate the sugar concentration in blood and increase the secretion of insulin, leading to its antidiabetic activity [123]. Piperine is also reported to be analgesic thanks to the blockage at the local level of prostaglandins [124]. Another property of piperine is its anticancer activity [121]. This power can be related to mechanisms such as cell cycle arrest and apoptotic power, as well as reductions in the levels of lipid peroxidation and carbonyl proteins, thus decreasing the time of growth of a tumor mass and increasing survival time [123]. Some researchers have also assessed the possibility of including piperine in drugs, as this may increase the effectiveness of advanced drug delivery systems [108].

Piperyline forms part of the alkylamide class and represents 1% of black pepper’s EO; it is also responsible for the pungency, even though it has a higher threshold level (5.1 nmol/cm^2^) than piperine (3.0 nmol/cm^2^) [74,125]. According to the literature, only a few papers focus on the biological activities of pure piperyline; in particular, Park et al. [126] reported that piperyline has biological effects associated with cell adhesion, migration, proliferation, and osteoblast differentiation, and they suggested the potential role of this alkaloid in the treatment of bone diseases.

Another characteristic compound of black pepper is β-caryophyllene, a bi-cyclic sesquiterpene of the terpenoid class that accounts for up to 30% of black pepper’s EOs [82,127]. In terms of sensorial aspects, β-caryophyllene stimulates the odorant receptors and is associated with the woody (patchouli, wood), spicy (clove, black pepper, powdery peppery), terpenic, and citrus–green (melissa) descriptors [120,128]. It can also stimulate taste, imparting a sweet note [120,129].

This molecule is a selective phytocannabinoid with various biological activities, such as anti-inflammatory, anticancer, and antimicrobial, and it can foster relaxation [130]. Its anti-inflammatory activity is related to its capacity to inhibit the main inflammatory mediators, such as interleukin and cyclooxygenase [131,132]. Some studies have also investigated the effects of β-caryophyllene on muscles, discussing the relaxing effects of this molecule as a possible therapy for the treatment of epilepsy [131]. β-caryophyllene also exhibits anticancer effects via the apoptosis-induced reduction in cancer cells and by decreasing the proliferation of pancreas and colon cancer cells [131]. Its activity against bacteria and fungi has also been reported [133].

Limonene is a monoterpene and represents up to 21% of black pepper EOs, thus exerting an important sensorial effect. It is generally associated with odor descriptors such as lemon, orange, citrus, and freshness (terpenic) [56,82].

In the literature, there are also some studies showing that this compound is important as a result of its biological activities. Limonene has shown anticancer activity; some studies have observed its effects on cell, animal, and epidemiological models, showing the capacity of this compound to alter cancer signaling pathways, causing apoptosis and preventing cancer proliferation [134,135,136]. This compound also influences the inflammatory response via its capacity to reduce the expression of cyclooxygenase and inducible nitric oxide synthase but also via the production of prostaglandin E2. It also decreases the presence of pro-inflammatory cytokines TNF-α, interleukin-6, and interleukin-1β [135,136]. Another property is its gastroprotective effect related to its capacity to stimulate the production and secretion of mucus and heat shock protein HSP70 [136]. Lastly, its antioxidant activity both in vitro and in vivo is related to its ability to scavenge for free radicals [135].

### 3.2. Cubeb Pepper (Piper cubeba *L.f.*)

*Piper cubeba* L.f. belongs to the *Piper* genus of the Piperaceae family, and it is commonly known as “cubeb pepper”, “tailed pepper”, or “Java pepper”, as it is native to Java, Borneo, Indonesia and India [4]. *P. cubeba* L.f. was first used as a traditional medicine and a spice in the Middle Ages; nowadays, the fruit has applications in cosmetics, food preservation, and perfumery [4,43,59,64]. In the fruit, the predominant components identified are piperine (0.1%) [77], (-)-cubebin (1–3%), (-)-hinokinin, and dihydrocubebin [4,43,63]. In terms of chemical classes, coumarins, polyphenolics, fatty acids, terpenoids, glycosides, anthraquinones, saponins, carbohydrates, and tannins have been found [3,4,19,29,30,108]. The secondary metabolites of cubeb pepper enhance the plant’s competitiveness in its environment [137] and are valued in the areas of pharmacology, cosmetics, food preservation, agriculture, and medicine. In herbal medicine, cubeb pepper products are used as treatment for gastrointestinal, respiratory, and renal disorders due to their analgesic and anti-inflammatory activities [3,29,59,138]. The edible part of cubeb pepper is the fruit, a globose berry of 6–8 mm in diameter that is smooth but wrinkled when dry, hard, and pedunculated, with a seed (globose) [4,30]. *P. cubeba* L.f. fruits have a spicy aromatic odor with a bitter, pungent, and persistent taste; the dried pericarps possess a grayish-brown color with the typical “tail” attached, giving this fruit the name “tailed pepper” [4,19,30]. The characteristic aroma of cubeb pepper comes from the essential oil compounds found within the cell walls of the pericarp. Some authors [4,139] have analyzed the aromatic volatile compounds of *P. cubeba* L.f. extracts via gas chromatography/mass spectrometry (GC/MS), identifying eugenol (33.95%), methyl eugenol (41.31%), β-cubebene (18.3%), and α-cubebene (4.1%) as the most abundant components in essential oils. Table 2 and Table S1 summarize the fruit’s main biological activities and the most relevant studies on cubeb pepper use and dosage, along with the principal results. 

**Figure 2:**
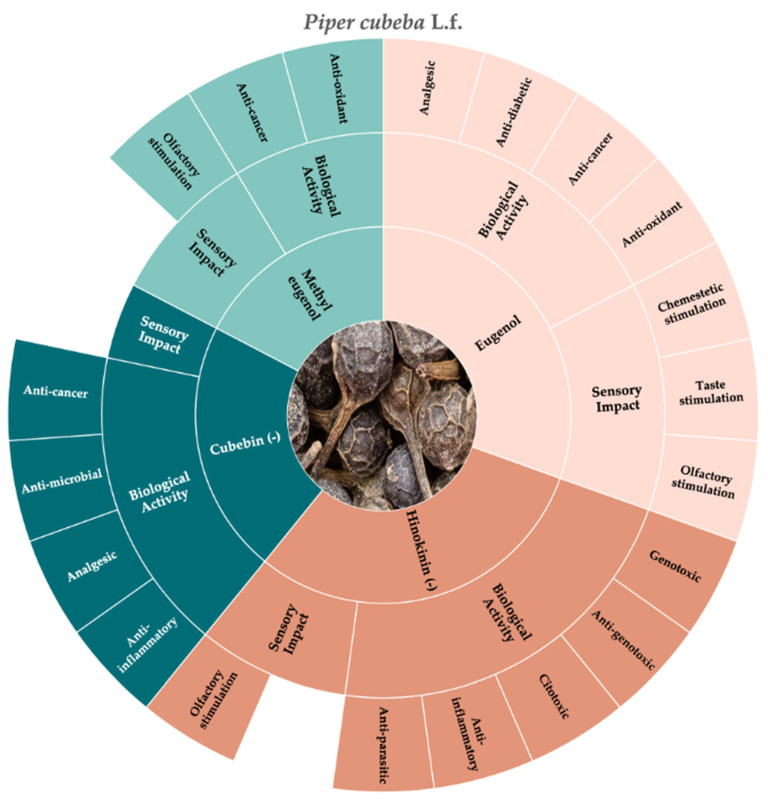
Key molecules and information gaps related to the sensory stimulation and biological activity of cubeb pepper (*Piper cubeba* L.f.).

According to the literature that we consulted, Figure 2 shows the molecules that best constitute both the chemosensory and the biological characteristics of cubeb pepper, which are eugenol, methyl eugenol, (-) cubebin, and (-) hinokinin.

Eugenol is a phenol compound of the phenylpropanoids class that is able to stimulate the odorant receptors associated with spicy (clove, cinnamon, allspice), herbal (woody, hay), honey, and smoky (phenolic, ham, bacon) odor descriptors [120,128,140,141]. Eugenol is also a chemesthetic compound evoking an oral sensation of innocuous warming; it is in fact responsible for the activation of the TRPV1, TRPV3, and TRPA1 receptors involved in pain nociceptive responses [118]. In addition to the sensory properties, eugenol exhibits a variety of biological activities. Firstly, it is known for its antioxidant power due to its ROS scavenging capacity [139,142,143].

Moreover, eugenol is reported to induce cancer cell apoptosis, increasing non-specific membrane permeability [142] against different cancer lines (colon, skin, and gastric carcinoma).

Its antidiabetic activity has also been reported, since eugenol inhibits α-glucosidases, the enzyme responsible for hydrolyzing carbohydrates into monosaccharides, preventing a rapid rise in blood sugar levels [142]. Finally, eugenol is known to be analgesic, since it can reduce the pain-related responses and is thus able to suppress the histamine response, stimulate periarterial sympathetic nerves, and inhibit prostaglandin synthesis [142].

Methyl eugenol is a phenylpropanoid [144] produced by the methylation of eugenol [145,146]. This compound represents 41.31% of the volatile compounds in the EOs of cubeb pepper. It is able to stimulate the odorant receptors associated with the spicy (clove, cinnamon, spice), herbal (vegetable), earthy (musty), and oily (waxy) odor descriptors. As a derivative of eugenol, it carries similar sensory and bioactive properties, such as antioxidant activity, especially against renal oxidative damage [139,142,147], and it shows anticancer activity against retinoblastoma cells [148].

Cubebin belongs to the dibenzylbutyrolactols lignan class [149] and is the most abundant lignan isolated from cubeb pepper fruit [4]. In the literature, there are no reports of cubebin exhibiting any sensory characteristics. On the contrary, several studies have identified the biological activities of cubebin. Some studies reported cubebin’s anticancer activity against diverse human cancer lines (colon and cervical carcinoma and myeloid leukemia) through cell-induced apoptosis [4,150]. Other authors have described the anti-inflammatory and analgesic activities of cubebin as related to reductions in the inflammatory process in edemas on rat paws, suggesting that cubebin presents a mechanism of action similar to non-steroidal drugs [4,150,151,152]. The antimicrobial and anthelmintic activities of cubebin have been demonstrated against different microbial populations, such as bacteria, fungi, protozoa, and mycobacteria [150,153]. Cubebin also exhibits a vasodilator action by releasing nitric oxide (NO), and some authors have proposed cubebin as an erectile enhancer and as a treatment for erectile dysfunction [150]. A cytotoxic study of cubebin highlighted a dose-dependent cytotoxic effect, with 280 μM as the maximum dose [154].

Hinokinin belongs to the dibenzylbutyrolactone lignan class [154,155]. It is capable of stimulating odorant receptors associated with oily (creamy, fatty) and sweet (caramel) odor descriptors [120]. Hinokinin exhibits anti-inflammatory effects on induced rat paw edema [66,150,154], and in *P. cubeba* L.f., it was isolated as a derivate from (-) cubebin, showing greater anti-inflammatory activity than (-) cubebin itself [66]. Hinokinin also presents antimicrobial activity, as well as antitrypanosomal and antiplasmodial activities. Its cytotoxic activity has been observed against different cancer lines, such as murine lymphocytic leukemia, human colon, and lung and breast adenocarcinoma, while it was shown ineffective against human pancreatic cancer, nasopharyngeal carcinoma, and gastric adenocarcinoma [154].

### 3.3. Long Pepper (Piper longum *L.*)

*Piper longum* L., commonly known as “long pepper”, belongs to the *Piper* genus as well. *P. longum* L. and *P. nigrum* L. are the species with the greatest medical, commercial, and economic significance [44]. *P. longum* L. is one of the older spices used in the Ayurvedic medicinal system in Indian culture [20,44]; the fruit is cylindrical, straight, or slightly curved, with a length of 5∓25 mm and a diameter of 2∓7 mm, with a light brown color [31].

Long pepper presents similar sensorial characteristics to black pepper (*Piper nigrum* L.), with a pungent bitter taste that produces numbness on the tongue [31]. The chemical compositions of *P. longum* L. EOs have been studied through GC/MS analyses, identifying β-caryophyllene (11.85% ± 1.96), α-humulene (6.25% ± 0.48), 1-heptadecene (11.03% ± 1.77), and N-heptadecane (2.4–11.9%) as major constituents [129,156].

The main secondary metabolites of *P. longum* L. are alkaloids; piperine is the most abundant alkaloid identified in the fruits and roots, with a concentration of 3–5%, followed by piperlongumine and piperlonguminine [44,108]. Piperine and piperlongumine are the main compounds responsible for the pungency of this fruit due to their antagonism to the nociceptive receptors present in the human mouth, with threshold values of 3.0 nmol/cm^2^ and 10.4 nmol/cm^2^, respectively [74,82]. In addition to alkaloids, in the extracts of *P. longum* L., terpenoids, steroids (stigmasterol and sitosterol), flavonoids, glycosides, essential oils, fatty acid, amide, lignans, and coumarins were identified [32,44,63]. Some of these compounds present bioactive activities, including anticancer, antidiabetic, immunomodulatory, hepatoprotective, renoprotective, antimicrobial, anti-inflammatory, analgesic, and cardioprotective activities (see Table 2). Many of these properties are related to the anti-inflammatory and antioxidant effects of the fruit, as well as its ability to modulate enzyme and signal pathways [4,26,67]. Table 2 and Table S1 summarize the main biological activities and relevant studies on long pepper use and dosage, as well as the principal results.

**Figure 3:**
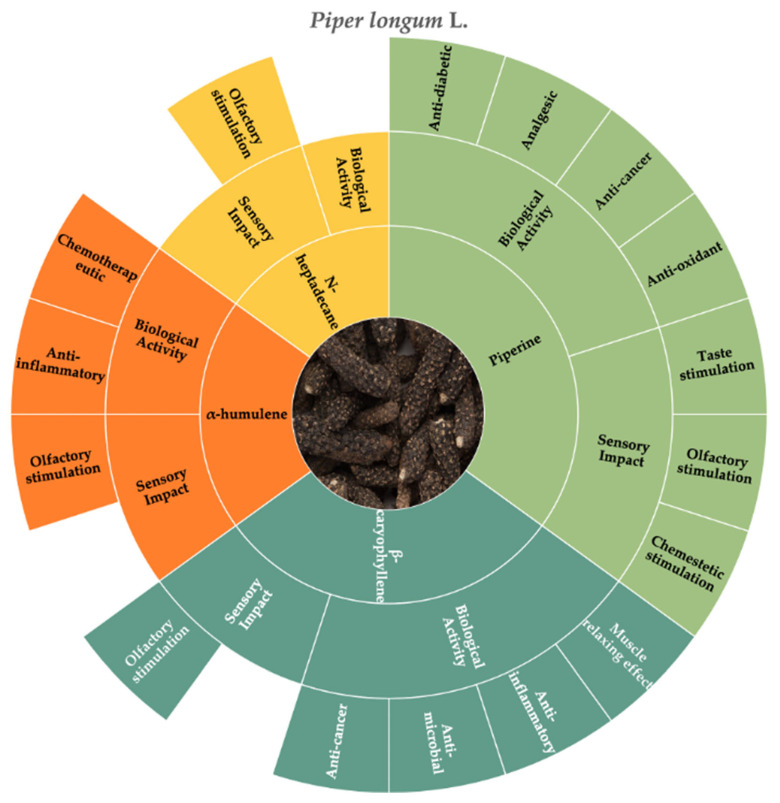
Key molecules and information gaps related to the sensory stimulation and biological activities of long pepper (*Piper longum* L.).

According to the literature consulted, the molecules that best represent the chemosensory and biological characteristics of long pepper are n-heptadecane, β-caryophyllene, α-humulene, and piperine (Figure 3).

N-heptadecane is an alkane from the organic compound class; it is one of the most abundant compounds found in long pepper essential oil, representing 2.4–11.9% [129,156]. N-heptadecane is capable of stimulating odorant receptors associated with odor descriptors such as caramel [157]; however, there is no biological activity associated with N-heptadecane that has been reported in the literature.

β-caryophyllene is one of the most abundant compounds in long pepper EO, representing 11.8% of the essential oil [127,129]. As previously described in Section 3.1, β-caryophyllene is responsible for the sensory traits, as well as the anti-inflammatory, anticancer, antimicrobial, and analgesic activities of long pepper [131].

Also known as α-caryophyllene, α-humulene represents around 6% of long pepper’s EO [129]. α-humulene belongs to the sesquiterpenes class and is responsible for odors described as woody and spicy–clove [120].

Several studies [133] have reported that this compound may be capable of imparting a few biological activities, including anti-inflammatory properties [158]. Additionally, α-humulene can be considered a chemotherapeutic, since it can inhibit the proliferation of hepatocellular carcinoma and induce apoptosis [159].

Piperine, which belongs to the alkylamide class, represents about 4% of the long pepper fruit EO [79]. As previously described in Section 3.1, piperine is responsible for certain sensory features (pungency and peppery odorants) and is associated with biological activities such as antioxidant, antidiabetic, analgesic, and anticancer in long pepper [74,82,121,160].

### 3.4. Pink Pepper (Schinus terebinthifolius *Raddi*)

Pink pepper, also known as aroeira, is classified as *Schinus terebinthifolius* Raddi, and it pertains to the *Schinus* genus of the Anacardiacee family [35]. Due to the use of pink pepper for its ornamental, culinary, and medical properties, its cultivation and distribution have become new sources of income for Brazil, which is today the main producer [34,46].

*S. terebinthifolius* Raddi fruits present a light pink to red color due to the presence of anthocyanins derivatives, cyaniding (which imparts red to magenta colors), and pelargonidin (responsible for orange to pink colors) [27]. The fresh pine and citrus aromas of pink pepper are associated with the presence of several terpenes [82]. In the mouth, pink pepper presents a sweet taste with a smooth and pungent peppery flavor [35,82,101,161,162]. The pungent flavor of pink pepper is not related to piperine but to the presence of terpenes and phenolic compounds that exhibit pungent, bitter, and astringent flavors [27,88].

Some authors [101,162] analyzed the aromatic volatile compounds of ripe pink pepper fruit extracts trough GC/MS, reporting an average content of 3% of EO. Due to their variety and growing regions, the presence and concentrations of compounds may vary [163]. However, the constant major volatile compounds in pink pepper EO are limonene (17.44–31.28%), δ-3-carene (28.71–30.37%), α-pinene (4.34–15.01%), and α-phellandrene (12.60–34.38%) [101,162,164,165].

The phytochemicals isolated from pink pepper are associated with diverse biological activities, such as antiviral, antimicrobial, anti-inflammatory, antidiabetic, anti-hypertensive [27], antitumor [46,47], analgesic [34], anti-allergic, and photoprotective [48]. Table 2 and Table S1 summarize the main biological activities and relevant studies regarding pink pepper’s use and dosage, along with the principal results.

**Figure 4:**
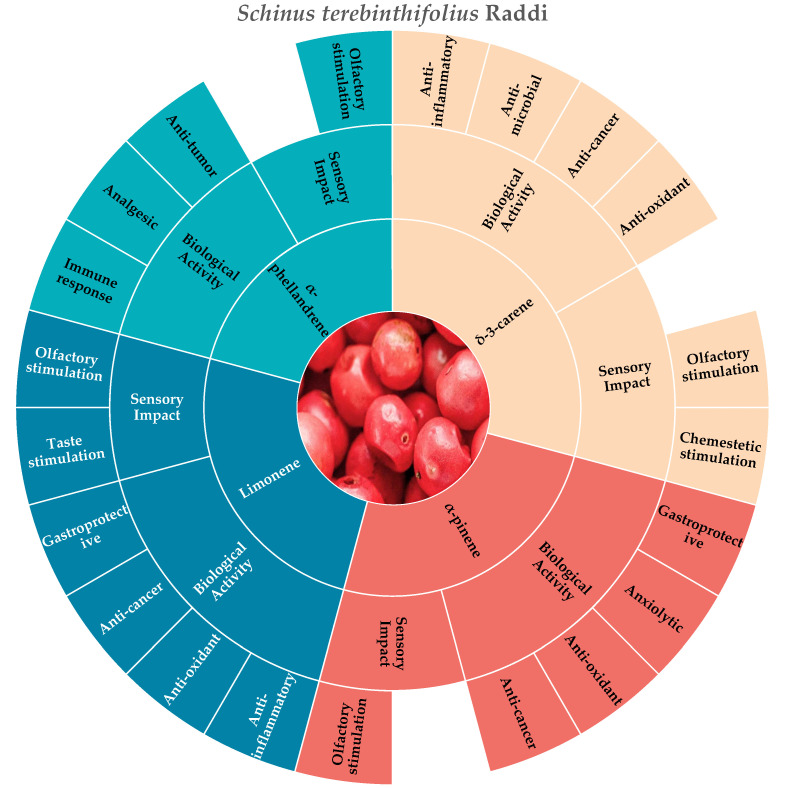
Key molecules and information gaps related to the sensory stimulation and biological activities of pink pepper (*S. terebinthifolius* Raddi).

According to the consulted literature, the molecules that best represent the chemosensory and biological characteristics of pink pepper are limonene, δ-3-carene, α-pinene, and α-phellandrene (Figure 4).

Limonene represents 17.44–31.28% of the EO of pink pepper [101,162]. Limonene is also present in black pepper EOs and shows interesting sensory and biological activities (anticancer, anti-inflammatory, antioxidant, and gastroprotective) [134,135,136] (see Section 3.1).

δ-3-carene is a bi-cyclic monoterpene from the terpene class; it constitutes 28.71–30.37% of pink pepper’s EO [101,162]. δ-3-carene is capable of stimulating odorant receptors associated with the terpenic (citrus, resinous, solvent), phenolic (medicinal), and cypress (herbal, pine, woody) odor descriptors [164]. In addition to sensory characteristics, δ-3-carene has biological properties, showing antioxidant effects via the reduction in free radicals [166], antimicrobial activity via the inhibition of *Bipolaris myadis* and *Rhizoctonia solani* [167], anticancer effects by reduction in the total growth of human tumor cell lines [73], and anti-inflammatory activity by reducing carrageenan-induced pedal edema in rats [132].

α-pinene is a bi-cyclic monoterpene from the terpene class; it represents 4.34–15.01% of the EO of pink pepper [101,162,164]. α-pinene activates olfactory receptors associated with terpenic (turpentine, woody), camphor (pine, fresh, herbal, cooling), and tropical odor descriptors [120]. α-pinene exhibits biological properties such as potent antioxidant effects by reducing the histological damage and myeloperoxidase activity in the rat lung and pancreas, as well as anti-inflammatory and anti-carcinogenic effects in the signaling process inhibiting inflammation via NF-κB and prostaglandin E1 [168,169,170].

α-phellandrene is a cyclic monoterpenoid and represents 12.60–34.38% of pink pepper’s EO [162,164,165]. α-phellandrene activates the odorant receptors associated with terpenic (citrus, turpentine, mint), spicy (peppery, black pepper), woody, and herbal (green) odor descriptors [120,128,171]. This monoterpene has been isolated and used as a pure molecule in many research studies, showing its antimicrobial, antioxidant, antinociception, antitumor, and anti-inflammatory activities [165]. α-phellandrene is a powerful anticancer compound that is active against various cancer types (lung, breast, prostate, liver cancer and leukemia) and promotes immune responses [165,171]. Its analgesic activity seems to be related to its capacity to regulate histamine, prostaglandin E2, and serotonin [165]. While α-phellandrene has beneficial effects, topical application may lead to skin irritation, and oral intake can cause nausea, vomiting, diarrhea, and intestinal issues. Therefore, more extensive research on its potential adverse effects is necessary [165].

### 3.5. Allspice (Pimenta dioica *L. Merrill*)

The genus *Pimenta* Lindl. consists of around 18 species, but the most famous one is the *Pimenta dioica* (Linn.) Merrill [12]. *P. dioica* (L.) Merrill is part of the Myrtaceae family and is known as “allspice” due to its complex spicy flavor reminiscent of a mixture of clove, cinnamon, and nutmeg [38]. Jamaica (West Indies) is the main distributor of *P. dioica* (L.) Merrill, representing around 70% of the world’s production [12,39,52].

Allspice presents a dark-green color when ripe, and it is brown when dried, with a complex aroma and flavor profile [12]. When the berry is crushed, its aroma resembles a blend of clove, cinnamon, and nutmeg [12,38,82]. The aromatic compounds of allspice EO have been analyzed through GC/MS, and an average content of 1.02 ± 0.8% of essential oil has been reported. Although variations in compounds and their contents have been found in the literature due to the fruit’s variability, the main compounds consistently identified in allspice EO are eugenol (60–90%), methyl eugenol (9–15%), β-caryophyllene (4.03%), and 1,8-cineole (1.86%), all associated with sensory traits [12,36,53,69,172]. In addition to its sensory characteristics, allspice is used as an aphrodisiac and as an agent to treat digestive disorders [38,69]. Its secondary metabolites present a wide spectrum of bioactivities, such as antioxidant [53], anti-inflammatory [12], and antibacterial [173], as reported in Table 2. In the Supplementary Materials Table S1 summarizes the relevant studies regarding allspice use and dosage and their principal results.

**Figure 5:**
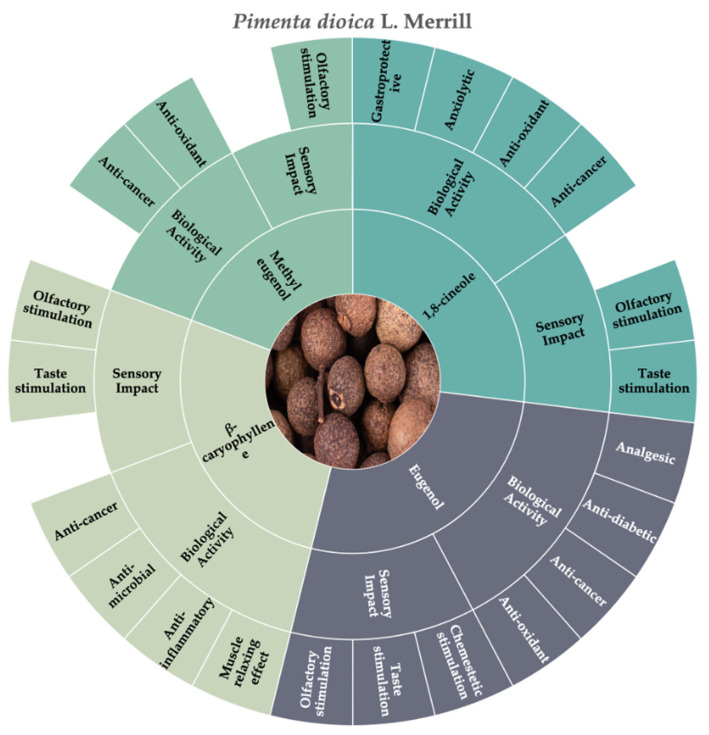
Key molecules and information gaps related to the sensory stimulation and biological activity of allspice (*P. dioica* (L.) Merrill).

According to the consulted literature, the molecules that best represent the chemosensory and biological characteristics of allspice are eugenol, methyl eugenol, β-caryophyllene, and 1,8-cineole (Figure 5).

According to the literature, eugenol represents 60–90% of the allspice essential oil, while methyl eugenol represents 9–15% [12,172]. Their odor descriptors and biological activities were reported previously in Section 3.2.

β-caryophyllene represents 4.03% of the allspice essential oil [172], and, as previously described in Section 3.1, it is capable of stimulating odorant receptors and is associated with many biological activities.

1,8-cineole, also known as eucalyptol, is a monoterpene cyclic ether that constitutes 1.86% of the essential oil present in allspice [172]. 1,8-cineole stimulates the odorant receptors associated with herbal (eucalyptus) and camphoraceous (medicinal, mint) odor descriptors [120,128]. 1,8-cineole presents biological activities that act via diverse mechanisms of signaling, such as an anti-inflammatory effect by reducing pro-inflammatory enzymes; it also has an antioxidant activity, exhibited by neutralizing reactive oxygen species (ROS), and an antimicrobial activity by inducing cell membrane permeability [174]. 1,8-cineole has been proposed for use as a treatment in neurological disorders due to its ability to traverse the blood–brain barrier [174].

### 3.6. Japanese Pepper (Zanthoxylum piperitum *DC.*) 

The genus *Zanthoxylum* (formerly spelled *Xanthoxylum*) belongs to the Rutaceae family and includes around 246 species, the most recognized species being *Z. piperitum* DC. and *Z. bungeanum* [40,41,84,175]. *Z. piperitum* DC., commonly known as “Japanese pepper”, “Japanese prickly ash”, or “sanshō” [40,60,85], is valued for the dry pericarp of its berry, which is used as a spice, while its fruit is used as a herbal medicine, and its extracts are commonly used as fragrances [41,58].

Japanese pepper (*Z. piperitum* DC.) presents two different colorations: green and red. The green is associated with unripe fruits harvested in spring, while the red is associated with fruits harvested in the fall [28].

Due to Japanese pepper’s unique organoleptic characteristics, many researchers have focused on the compounds responsible for its sensory traits and their mechanisms of action. Organoleptically, *Z. piperitum* DC. has a strong and pleasant aroma with woody, spicy, and citrus notes due to the presence of volatile compounds like limonene, α-pinene, *β*-phellandrene, and citronellal [28,56,176], these being the molecules that are most present and representative of the Japanese pepper’s aroma. Its most unique trait is the chemesthetic sensation of numbness, buzzing, and tingling that it can cause in the mouth when it is eaten [40,41]. These sensations are caused by the alkylamide compounds α-sanshool, β-sanshool, hidroxy-α-sanshool, and hidroxy-β-sanshool [40,41,84].

As widely reported in the literature [40,60,61,85,177], *Z. piperitum* DC. has shown many biological activities, such as antioxidant, digestant, and anticancer activities, and the ability to reduce lipids and exert antidiabetic effects (see Table 2). In the Supplementary Materials Table S1 summarizes the relevant studies regarding Japanese pepper’s use and dosage, along with the principal results. 

**Figure 6:**
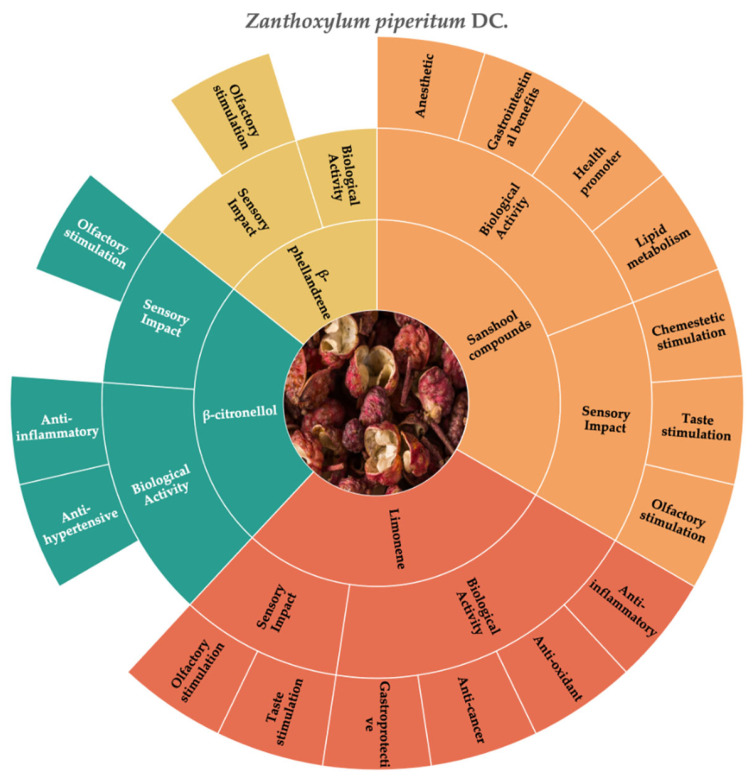
Key molecules and information gaps related to the sensory stimulation and biological activity of Japanese pepper (*Zanthoxylum piperitum* DC.).

According to the literature that we consulted, the molecules that best represent the chemosensory and biological characteristics of Japanese pepper are limonene, β-phellandrene, sanshool compounds, and β-citronellol; Figure 6 reports their sensory impacts and biological activities. 

Japanese pepper is characterized by the presence of the “sanshool” class compounds. The most prevalent sanshools are α-sanshool, β-sanshool, hydroxyl-α-sanshool, and hydroxyl-β-sanshool.

These compounds share a common structure characterized by three parts: the “head”, represented by the vanillyl group; the “neck”, where the amide bond is located; and the “tail”, represented by an aliphatic chain [41,56,178].

Their structure allows the activation of nociceptive receptors present in the mouth, causing different chemesthetic perceptions, such as burning, tingling, and numbness, depending on the molecular structure. Luo et al. [41] have proposed mechanisms to explain how these substances can cause the feeling of numbness. These compounds can depolarize the transient receptor potential (TRP), particularly for TRPV1 and TRPA1, which is found on the sensory nerves in the mouth. This depolarization can cause Ca^2+^ influx and induce an internal current, causing the feeling of numbness. Another explanation for this sensation could be the inhibitory effects of compounds on the two-well potassium channels, such as KCNK3, KCNK9, and KCNK18; this action could produce an anesthetic effect due to the activation of sensory neurons [41,56].

These molecules are distinguished by a few bonds or substituents that allow them to stimulate different sensations. The alkylamide α-sanshool is responsible for burning, tingling, and numbness sensations, with a CTL of 13 μg/mL; the alkylamide β-sanshool is responsible for tingling (and the bitter taste), and it has a CTL of 14 μg/mL; the hydroxyl-α-sanshool is associated with burning and tingling sensations, with a CTL of 38 μg/mL; the hydroxyl-β-sanshool is responsible for numbness and astringency (and the bitter taste), with a CTL of 78 μg/mL [40,41,84,86].

The contents of these compounds vary with their ripeness index. The α-sanshool content shows a trend of decreasing with increasing maturity and ranges from 4.7 ± 0.1 mg/g dry weight (DW) to 12.0 ± 0.1 mg/g DW. On the other hand, the β-sanshool (ranging from 0.1 ± 0.0 mg/g DW to 1.1 ± 0.1 mg/g DW), hydroxyl-α-sanshool (ranging from 20.0 ± 1.2 mg/g DW to 52.3 ± 3.2 mg/g DW), and hydroxyl-β-sanshool (ranging from 0.6 ± 0.0 mg/g DW to 5.6 ± 0.6 mg/g DW) compounds increase in content with the maturation of the fruits [86].

Despite many studies devoted to fully describing the chemesthetic activities of the sanshool compounds, to the best of our knowledge, their bioactive effects still need to be analyzed in depth.

According to the literature, hydroxyl-α-sanshool has several effects, including a positive impact on the gastrointestinal system, strong salivatory effects, the activation of TRPV channels, the stimulation of somatosensory neurons via the inhibition of potassium channels, and local anesthetic properties [22,84,85]. Animal studies have also suggested that hydroxyl-α-sanshool may improve lipid metabolism, highlighting its potential use as a therapeutic and health-promoting agent [22,85].

Limonene is one of the compounds that Japanese pepper shares with pink and black peppers, as analyzed above, and its content in *Z. piperitum* DC. EO is about 5.48% [176]. Its sensorial impact and biological activities are described above in Section 3.1. According to the available literature, limonene is responsible for some of the biological activities associated with Japanese pepper, such as its anticancer, anti-inflammatory, antioxidant, and gastroprotective activities [134,135,136].

*β*-citronellol is an acyclic monoterpenoid present in Japanese pepper EO at 10.32% [176]. It is the molecule responsible for odor profiles such as citronella oil, rose leaf, and rose petal [120]. In addition to its sensory properties, *β*-citronellol is also studied for its biological activity, but only a few studies on this are available in the literature. According to Ribeiro-Filho et al. [179], *β*-citronellol can exhibit an anti-hypertensive effect due to its vasodilatory abilities. Moreover, a study conducted by Iqbal [180] evaluated the potential anti-inflammatory activity of *β*-citronellol using both in vitro and in vivo models, reporting that this compound could be a potential therapeutic agent used in the treatment of inflammatory conditions.

In *Z. piperitum* DC. EO, one other compound that is important for the sensory profile characterization is *β*-phellandrene, the content of which is about 29.39% [176]. This compound is a monoterpene associated with the odor descriptor of mintiness [120,128]. According to Yamasaki et al. [28], despite the high content of *β*-phellandrene, its contribution to the sensory profile of this fruit is small, while the ratios of geranyl acetate, citronellal, and limonene are more relevant. Based on our current knowledge, no specific papers related to the bioactive properties of *β*-phellandrene extracted from *Z. piperitum* are available in the literature.

## 4. Review Methodology

A data search was carried out using diverse digital research libraries and bibliographic databases (e.g., Scopus, Science Direct, PubMed, and Google Scholar) over a period covering the last 15 years, with the aim of achieving the greatest coverage of relevant papers and exploring the progress of the spices’ nutraceutical use. Initially, we focused our attention on reviews in order to critically select the most important recent publications. Then, taking the base of documents selected from the predetermined time frame, we included older literature sources, which helped to widen the topic description.

Data collection was performed using the OR and AND operators, focusing on documents in which the keywords “*Piper nigrum* L.”, “*Piper cubeba*”, “*Schinus terebinthifolius*”, “*Piper longum*”, “*Pimenta dioica* L.”, “*Zanthoxylum piperitum*”, “black pepper”, “cubeb pepper”, “pink pepper”, “long pepper”, “allspice”, and “Japanese pepper” were present individually or in combination. The data were collected from articles and reviews, which were subsequently evaluated independently by four authors. The eligibility criteria included relevant studies on the fruits’ biological effects, as well as their consumption and chemesthesis, with any disagreement resolved by discussion. The articles and reviews considered the connections between key compounds and their effects on odorant receptors [181], taste receptors [182], and chemesthetic perception [183]. A total of 184 papers were finally selected for the review. Since the data collected highlight both the benefits and detriments of the six spices, a SWOT analysis was considered to evaluate their importance in terms of sensory and health effects. SWOT is an acronym for strengths, weaknesses, opportunities, and threats [184].

## 5. Conclusions

The bioactive, chemical, and sensory properties of spices vary significantly due to factors such as cultivation practices, growing environments, and societal culture, including farmers’ beliefs and traditional methods employed tied to geographical origin, thus complicating the efforts to assess or compare their quality, composition, and health effects.

Thanks to the work carried out as part of this review, it was possible to identify, for each of the six selected spices commonly referred to as “pepper”, certain key molecules that can be associated with both the strengths and weaknesses of each spice. These molecules give each spice its distinctive traits in terms of both sensory characteristics and nutraceutical potential. Spices are rich in chemosensory-active compounds (CaCs), which enhance the sensory impacts of foods by stimulating taste receptors and olfactory senses, as well as the trigeminal nerve, contributing to properties like pungency, tingling, and warming. However, the high variability of CaC concentrations in each spice necessitates a careful evaluation of their sensory effects, both individually and in combination with various food matrices. This variability makes it extremely challenging to accurately estimate the sensory profiles based on data from the literature alone without conducting an actual sensory evaluation. In this context, there is also a notable lack of studies aimed at establishing sensory characterization protocols for spices. Concerning the biological effects of the spices under consideration, a significant heterogeneity was observed, as studies often focus on different types of extracts (EOs or individual compounds) and use varied methodologies, including in vivo and in vitro tests on diverse target organisms. Research on the effects of spices in their natural form is scarce and is further limited by the variability in spices’ compositions, particularly in terms of the concentrations of bioactive compounds. To better understand their bioactive properties and develop innovative therapeutic approaches, more targeted and standardized studies are needed.

## Figures and Tables

**Table 1:** List of spices selected based on common culinary use and being referred to as “pepper”.

Botanical Name	Family	Distribution Area	Common Name	Edible Organs	Method of Production
*Piper nigrum* L.	*Piperaceae*	Hainan, Yunnan, and Guangdong in China and Europe	(*eng*) black pepper, (*fra*) poivre noir, (*esp*) pimienta negra, (*deu*) schwarzer pfeffer, (*ita*) pepe nero	Fruit and bark	Berries are harvested at early ripening when they turn yellow. Then, berries are washed in hot water, and finally, they are sun-dried or dried by artificial methods [16,17,18].
*Piper cubeba* L.f	*Piperaceae*	Sri Lanka, Sumatra, Malaysia, Southern Borneo, and Java	(*eng*) cubeb pepper, (*fra*) poivre cubèbe, (*esp*) pimienta cubeba, (*deu*) kubeben pfeffer, (*ita*) pepe cubebe	Fruit	The fruits are harvested by hand when ripe and separated from spikes. Then, the berries can be directly dried or immersed in water to remove the pericarp, and afterwards, they are dried for 3–4 days [19].
*Piper longum* L.	*Piperaceae*	India, Malaysia, Indonesia, Singapore, Sri Lanka	(*eng*) long pepper, (*fra*) poivre long, (*esp*) pimienta larga, (*deu*) langer pfeffer, (*ita*) pepe lungo	Dried infructescence and leaves	The infructescence is harvested before ripening when the color is blackish green. Subsequently, the berries are dried in the sun for about 4–5 days [20].
*Schinus terebinthifolius* Raddi	*Anacardiacee*	Central and North America, Europe, Asia, and Africa	(*eng*) pink pepper or false pepper, (*fra*) faux poivrier, (*esp*) pimienta de brasil o pimienta rosada, (*deu*) rosa pfeffer, (*ita*) pepe rosa	Fruit	The berries are harvested manually once they have reached maturity and then dried [21].
*Pimenta dioica* (L.) Merrill	*Myrtaceae*	West Indies (Jamaica) and Central America (Cuba, Mexico, Brazil, Honduras, Guatemala, Belize)	(*eng*) Jamaica pepper or allspice, (*fra*) poivre de la jamaïque, (*esp*) pimienta de jamaica, (*deu*) jamaika pfeffer, (*ita*) pepe garofanato	Fruit and leaves	The berries harvested are left for up to 5 days in sacks to ferment. Then, they are dried from 5 to 10 days, depending on the weather, until the moisture content is about 12% [16].
*Zanthoxylum piperitum* DC.	*Rutaceae*	Japan and Korea	(*eng*) Japanese pepper, Japanese prickly ash; (*fra*) poivre japonais, (*esp*) pimienta japonesa, (*deu*) japanischer Pfeffer, (*ita*) pepe giapponese	Fruit and leaves	Sansho fruits are harvested when ripe. After harvesting, the fruits are dried, and the seeds are removed, leaving dried pericarps [22].

**Table 2:** Classification of biological effects associated with the selected spices.

TARGET APPARATUS	SPICES					
	*Piper nigrum* L.	*Piper cubeba* L.f	*Piper longum* L.	*Schinus terebinthifolius* Raddi	*Pimenta dioica* L. Merrill	*Zanthoxylum piperitum* DC.
**Metabolic effects**	Anti-thyroid; treatment of obesity [25]	Cardiovascular; antidiabetic; hypocholesterolemic; treatment of renal disorders; anti-urolithiatic [3,4,19,29,30]	Antidiabetic treatment of insomnia and pyrexia; anti-hyperlipidemic; anti-obesity [4,26,31,32,33]	Antidiabetic; anti-hypertensive [27,34,35]	Antidiabetic; cardiovascular; hypotensive activity; increase in body weight [36,37,38,39]	Decreased lipids, appetite stimulant [40,41]
**Protective effects on tissue and organs**	Antioxidant; hepatoprotective; anti-apoptotic [25,42]	Treatment of enteritis; antioxidant; hepatoprotective; renoprotective; prophylactic agent; anti-ulcer [3,4,19,30,43]	Antioxidant; hepatoprotective; renoprotective; anti-arthritis [26,31,32,33,44]	Antioxidant; cicatricial agent; hepatoprotective; treatment of urinary infections, rheumatism, and wounds; protective against doxorubicin-induced cardiotoxicity [27,35,45,46,47,48,49,50,51]	Antioxidant; as treatment for rheumatism [12,36,37,38,52,53,54,55]	Antioxidant; anti-osteoclastic; anti-osteoarthritic [40,56,57,58]
**Effect on respiratory system**	Respiratory disorder [25]	Anti-asthmatic [4,19,29,30,43,59]	As treatment for respiratory disorder (bronchitis, asthma) [26,31,32,44]			
**Effect on gastrointestinal system**	Anti-diarrheal; anti-colon toxin; gastric ailments [25]	Treatment against dysentery, diarrhea [4,19,29,30,43,59]	As treatment for dysentery and stomach disease [26,31]		As treatment for diarrhea; cramp; indigestion; flatulence; nausea; carminative [36,37,38,39]	Digestant; induces ileum and distal colon contraction; gastrointestinal protection; relaxes gastric body; as treatment for digestive disorders [56,60,61]
**Effect on nervous system**	Anti-depressant; neuroprotective; antispasmodic; analgesic [4,25,62]	Vasorelaxant; modulator of human monoamine; GABA transporter; anti-depressant; anti-nociceptive; analgesic neuroprotector [4,12,19,30,63,64,65]	Neuroprotective; anti-stress; anti-Parkinson and Alzheimer; nootropic; anti-convulsant; analgesic [26]	Analgesic [27,34]	As treatment for neuralgia; sedative; spasmolytic; analgesic; anesthetic; anti-depression; anti-stress [12,37,38,39,54]	
**Effect on immunologic system**	Anti-pyretic; immunomodulatory; anti-inflammatory; intermittent fever [25,42,62]	Antiproliferative; anti-inflammatory; immunomodulatory; anti-ulcer [4,19,29,30,59,64,65,66]	Immunomodulatory; anti-inflammatory [26,32,44,67]	Anti-inflammatory; anti-allergic; antiproliferative [27,34,35,47,50,51]	Anti-inflammatory; antiseptic [37]	Anti-inflammatory [40,56,58,61]
**Enhanced activities**	Pancreatic amylase, chymotrypsin activation, protease, lipase [25]	Wound healing [4,19]	Circulatory stimulant, vasorelaxant [26,33]		As stimulant in digestive disorders (purgatives) [37,38]	Enhanced transdermal drug penetration [40]
**Response to biotic factors**	Insecticidal; larvicidal; pesticidal; antibacterial; antifungal [4,25,42,62]	Anti-protozoal; trypanocidal; antiviral; as treatment against syphilis gonorrhea; leishmanicidal; molluscicidal; insecticidal; acaricidal; anti-amebic; antibacterial; antifungal; antiparasitic [4,19,29,30,59,63,64,65]	Antibacterial; mosquito larvicidal; acaricidal; anti-amoebic; antifungal; antimicrobial; anthelmintic [26,31,33,44,67]	Antiviral; antimicrobial; antifungal; insecticidal; antibacterial; protective against multi-drug-resistant strains [27,35,47,48,50,68]	Antimicrobial; acaricidal; antifungal; nematicidal; inhibits *Leishmania amazonesis*; antibacterial; anti-SARS-Cov 2; antiparasitic [12,36,37,38,52,55,69,70]	Antimicrobial; vermicide; antiviral; as herbicide barrier [40,60]
**Effect on genomic expression**	Anti-mutagenic; inhibits transcription; inhibits cytochrome; anti-metastatic; antitumor	Anti-mutagenic; cytotoxicity; anticancer; antitumor; genotoxic [4,19,29,30,59,63,64,65]	Anticancer and tumor [4,26,31,32,33,44]	Antitumor [50]	Anticancer [37,38]	Control proliferation of osteosarcoma [58]
**Adverse effects**	Anti-spermatogenic; interactions with medical products; may affect pregnant or breastfeeding women; EO could be considered as irritant	Genotoxic at 1.5 g/kg; may irritate the gastrointestinal tract and kidneys; cytotoxic at 280 mM of (-) cubebin; irritant [19,30,64]	Emmenagogue; anti-conceptive; infertility action; ACE inhibitor; increases the weight of lungs and spleen [26,31,33]	May cause allergies, mutagenic properties [45]	Some EOs could be cytotoxic, irritant, corrosive, and phytotoxic [12]	
**Other effects**	Ciprofloxacin potentiator; enhances the bioavailability of some nutrients like vitamins, β-carotene, and selenium [25]	Anti-platelet-activating factor (PAF); CYP3A4 inhibitor; cytochrome P450 inhibitor; melanogenesis activity [4,30,63]	Aphrodisiac; anti-angiogenic; anti-platelet; anti-ulcer; photoprotective [26,33,67]		Cytoprotective [64]; aphrodisiac; digestive stimulant agent; rubefacient agent [38]	Prevention of toothache; use as skin-lifting agent [40,56]

## Supplementary Materials

The following supporting information can be downloaded at: https://www.mdpi.com/article/10.3390/molecules30091891/s1. Table S1: Relevant studies regarding the use, dosage, and principal results of the compounds and spices selected [4,12,25,26,27,29,34,36,38,40,41,43,45,47,51,52,57,58,68,107,108,111,119,145].
