# Supplementary material for: “Pepper”: Different Spices, One Name—Analysis of Sensory and Biological Aspects
Source: Molecules. 2025 Apr 24;30(9):1891. doi: 10.3390/molecules30091891 (PMC12073208; doi:10.3390/molecules30091891)
Supplement: Supplementary file 1 [file molecules-30-01891-s001.zip › molecules-3418517-supplementary.pdf]

Supplementary Table S1. Relevant studies regarding use, dosage and principal results of compounds and spices selected.

Botanical name	Application mode/product presentation	Model organism	Dosage	Adverse Reactions / Results	Reference
Piper nigrum L.	Suspended, dissolved in vehicle, bolus administration	Male and female Sprague- Dawley rats	5-100 mg/kg bw/day of piperine	Disturbance of spermatogenesis and of maternal reproductive, embryotoxic effects and interactions with medical products	[25]; [108]
	Oral administration	Rats	170 mg/kg b/w of piperine	96–97% of the administered piperine dosage was absorbed	
	Powder from the fruits of *P. nigrum*	Human adults (≥18 years old)	250-420 mg per day	Interactions with medical products<br>May affect pregnant or breastfeeding women.	
	Piperine isolated	Human adults (≥18 years old)	Maximum dose per day 14 mg		
	Essential oil of *P. nigrum* and Supercritical extract of *P. nigrum* and oleoresin of *P. nigrum*	*P. nigrum* oleoresin for use in all animal species and a supercritical extract for use in dogs and cats	Use levels in food and beverages in the range of 3 mg/kg up to 206.4 mg/kg	The essential oil under assessment should be considered as irritant to skin and eyes, and as a dermal and respiratory sensitizer.	[107]; [145]
Schinus terebinthifolius Raddi	Oral administration and direct additive to food	*In vitro* and *In vivo* in male Wister rats	25-100 mg/kg bw of polysaccharides extracted from *S. terebinthifolius* Raddi and *S. molle* L.	May cause allergies in sensitive people even without direct contact with its leaves and fruits	[27]; [34]; [47]; [68]

	Extract of *S. terebinthifolius* fruits	*In vivo* with male albino rats	50 mg/kg body weight for 9 weeks	Inhibits activity of HMG-CoA reductase, an enzyme responsible in the metabolization of the cholesterol synthetic	[51]
	Simplex syrup extract	*In vitro* with *E. coli* strains and *Salmonella*	Dried bark decoction at a concentration of 20 mg/ml	Stem bark decoction showed mutagenic properties	[45]; [111]
Piper cubeba L.f.	Methanol extract	*In vivo* in female Wister rats	2,000 mg/kg body weight	Safe up to a maximum dose of 2,000 mg/kg body weight	[4]
	Essential oil	Male Wistar rats	50-3,000 mg/kg body weight	Showed no mortality nor overall behavioral alteration	
	Methanolic crude extract	Mouse breast cancer cell lines	IC_{50} value ≤20 µg/mL incubated for 7 days	Exhibited cytotoxic activity against breast cancer cells and normal breast cells and lower toxicity against normal fibroblast cells	[29]
Pimenta dioica L. Merrill	Essential oil of *P. dioica*	*In vitro* and *in vivo*	Highest concentration of *P. dioica* EOs allowed in food: 0.025%	Some bioactive compounds of EOs could be cytotoxic, irritant, corrosive, and phytotoxic	[12;38];
	Essential oil of *P. dioica*	Female BALB/c mice	Maximum single dose of 30 mg/kg of essential oil of *P. dioica*	Increase of body weight	[36]
	Ethyl acetate extract of leaves of *P. dioica*	*In vitro* SARS-CoV-2 main protease (Mpro)	10 mg/mL to 1 ng/mL of each isolated bioactive compounds	No toxicity tests have been carried out.<br>Rutin, gallic acid and chlorogenic acid showed anti-SARS-CoV-2 activities. Ferulic acid and rutin exhibited anti-inflammatory activities	[52]

Piper longum L.	*P. longum* fruit ethanol and hexane extract	Female rats	200 mg/kg (*P. longum* ethanol extract) from days 1–7 post coital, p.o.<br>20 mg/ml of *P. longum* hexane extract	The fruits present anti-fertility activity, its use during pregnancy and lactation should be avoided	[43]; [47]
	Ethanol extract of *P. longum* fruits	*In vivo* in mice	Acute dosages of 0.5, 1.0, and 3 g/kg for 24 hr and chronic dosage of 100 mg/kg/day for 90 days	Increased the weight of lungs and spleen	[26]
	Methanol extract of fruit - piperine	Dalton's lymphoma ascites (DLA) and Ehrlich ascites carcinoma (EAC) cells, L929 cells, DLA and EAC induced Balb/C mice	1.14 - 10 mg /dose /animal	100% toxicity (DLA and EAC cells), cytotoxicity (L929 cells), tumor development in DLA cells-induced mice ↓, life span in EAC cell-induced mice ↑	
Zanthoxylum piperitum DC.	*Z. piperitum* gel	7 weeks aged Sprague-Dawley male rats (250 ± 50 g)	1, 10 and 100 μg/mL dose-dependent of *Z. piperitum*	*Z. piperitum* inhibited alveolar bone loss and maintained periodontal structures via regulation of bone remodeling	[40]; [57]
	Ethanol extract of *Z. piperitum* via oral route	5 weeks aged female ICR mice underwent ovariectomies	1, 10 and 100 μg/mL for 6 weeks of *Z. piperitum*	*Z. piperitum* exerts its inhibitory effect against bone resorption by regulating RANKL-mediated c-fos/NFATc1/NF-κB in osteoclast	[58]
	Hydroxyl-α-sanshool	Taste testing in humans and extracellular nerve recordings in Sprague–Dawley rats (250–370 g)	5μl at 10 mg/ml in 70% ethanol for taste testing	Causes a tingling sensation<br>Could activate trigeminal neurons, thereby stimulating the sensory neurons	[41]; [119]
